# Improving Network Structure can lead to Functional Failures

**DOI:** 10.1038/srep09968

**Published:** 2015-05-19

**Authors:** Jan Philipp Pade, Tiago Pereira

**Affiliations:** 1 Humboldt University of Berlin, Institute of Mathematics, Unter den Linden 6, 10099 Berlin, Germany; 2 Department of Mathematics, Imperial College London, London SW72AZ, United Kingdom; 3 Institute of Mathematical and Computer Sciences, University of Sao Paulo, Sao Carlos, Brazil

## Abstract

In many real-world networks the ability to synchronize is a key property for their performance. Recent work on undirected networks with diffusive interaction revealed that improvements in the network connectivity such as making the network more connected and homogeneous enhances synchronization. However, real-world networks have directed and weighted connections. In such directed networks, understanding the impact of structural changes on the network performance remains a major challenge. Here, we show that improving the structure of a directed network can lead to a failure in the network function. For instance, introducing new links to reduce the minimum distance between nodes can lead to instabilities in the synchronized motion. This effect only occurs in directed networks. Our results allow to identify the dynamical importance of a link and thereby have a major impact on the design and control of directed networks.

Our everyday life depends on network synchronization at various levels. In power grids, power stations must keep a proper synchronization to avoid energy supply disturbances and blackouts^1^. Sensor networks rely on synchronization among sensors to transmit information^2^. In the brain, epileptic seizures and Parkinson’s diseases are a strong manifestation of synchronization^3^. Consensus formation is another example of synchronized activities^4^,^5^,^6^,^7^. These complex systems are modeled by networks with diffusive interaction, that is, the interaction between any two coupled elements depends on the difference of their states. So far, research efforts to understand the influence of connectivity on the dynamics have focused on undirected diffusive networks. For instance, it is known that increasing the homogeneity or the number of connections at random enhances synchronization^8^,^9^,^10^,^11^,^12^,^13^.

Networks found in nature are often *directed* and *weighted*. For example, electrical synapses in neuron networks have asymmetric conductance^14^, which makes the underlying network directed. Recent work has provided sufficient conditions to guarantee the stability of synchronization in such networks in terms of the network structure and nature of the interaction^15^,^16^,^17^. And although understanding the impact of structural modifications, such as changing weights and adding or deleting links on synchronization is fairly well understood for undirected networks, it remains an open problem for directed networks.

In this letter, using synchronization as a paradigm for network function, we show that improving the network connectivity structure can lead to a functional failure. This phenomenon has dynamical origins. Namely, in directed networks the structural improvement has a suppressing effect in the network spectrum leading to the onset of instabilities associated with the synchronous motion. Furthermore, we identify a class of links for which increasing the weights enhances synchronization. Our results provide a way to understand the dynamical importance of links in directed networks and to develop strategies to avoid functional failures when improving the network structure.

## Results

We consider directed networks of identical elements with diffusive interaction. The theory we develop here is general and can include networks of non-identical elements with minor modifications^16^. The network dynamics is described by


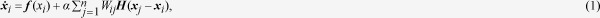


where 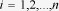, 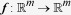 is smooth, 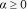 is the overall coupling strength, and the matrix 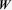 describes the network structure, i.e., 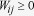 measures the strength of interaction from node 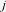 to node 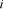. We assume that the network solutions are bounded and the local coupling function 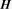 is smooth satisfying 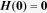. This last condition guarantees that the synchronous state 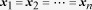 is a solution of the coupled equations for all values of 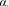 The overall coupling strength 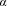 represents the fixed energy cost per connection.

**Figure 1:**
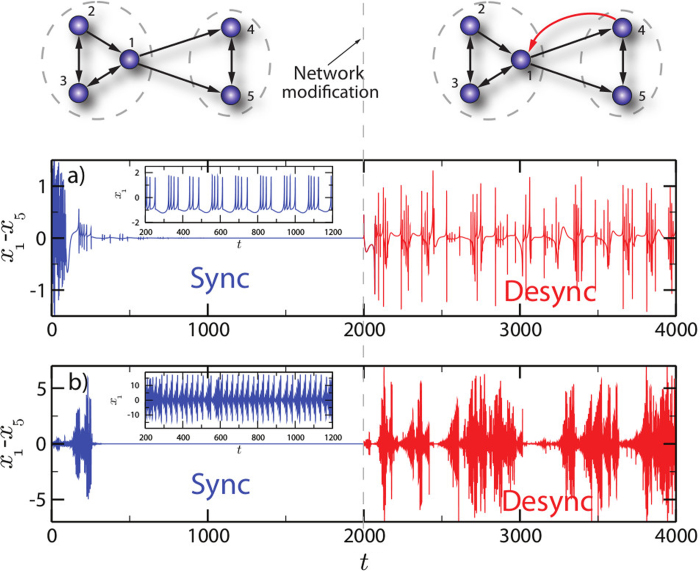
Improving connectivity leads to desynchronization. The figures show simulation results for the networks on top. All links in black have weight one. In the main plots we show the difference of the first component of *x*_1_ and the first component of *x*_5_. In **a**) the node dynamics is given by Hindmarsh-Rose (HR) neurons, and in **b**) by Rössler dynamics. The global coupling *α* is chosen such that the nodes synchronize chaotically for the original network. This can be seen in the main plots for times until *t* = 2000 in blue. The introduction of the new link 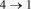 with weight 0.4 at time *t* = 2000 leads to a destabilization, displayed in red. The insets show the time series of a single node. For the HR neurons we consider a chaotic bursting mode and for the Rössler dynamics a chaotic state.

Here, we consider *weakly connected* networks, that is, when ignoring the link’s directions the network is connected. A directed network is *strongly connected* if every node can be reached by every other node through a directed path. Of course, a strongly connected network is also weakly connected. If a directed network is not strongly connected the links can be partitioned into two different classes: links belonging to some strongly connected subnetwork, called *strongly connected component*, and links belonging to some *cutset*. A cutset is a set of links which point from one strongly connected component to another^18^. In physical terms, this situation corresponds to a master-slave configuration. In the top left inset of Fig. 1 we show a network composed of two strongly connected subnetworks indicated by the gray dotted ellipses. The cutset consists of the links 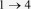 and 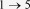, connecting the two components.

The smaller strongly connected component does not influence the larger component as there are no links from the smaller to the larger component. Nonetheless, the network still supports stable synchronous dynamics. The particular effects of directed networks come into play once we try to improve the network structure. Introducing a new link pointing from node 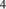 to node 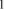 improves the connection structure significantly in the sense that the whole network is now strongly connected: any two nodes in the network are connected by a directed path. However, this structural improvement has fundamental consequences for the dynamics: the synchronous state becomes unstable.

**Figure 2:**
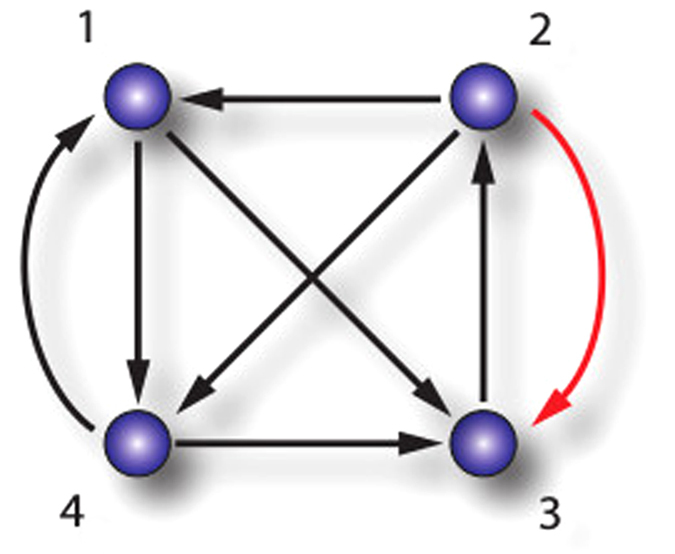
Desynchronization in a strongly connected network: Strengthening the link in red can lead to synchronization loss. Initially, all the links have weight one, however, increasing the weight on the link 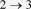 decreases the spectral gap. On the other hand, increasing weights on the links 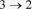, 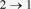 and 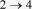 increases the spectral gap. Furthermore, introducing the new links 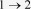 and 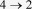 with a weak coupling strength also increases the spectral gap.

An important remark is that the effect of suppressing synchronization is not restricted to the master-slave configuration. The effect has nontrivial origins. For instance, except for the links 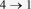 and 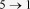, all other possible links opposite to the cutset (for instance 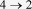) will make the synchronous state more stable. Moreover, synchronization loss can also be observed in strongly connected networks, if links are added or reinforced (see Fig. 2 and explanation below).

We illustrate this effect with two different classes of node dynamics, namely, Hindmarsh-Rose neurons in the chaotic bursting regime^19^ as shown in the inset of Fig. 1a), and chaotic Rössler oscillators^20^, see inset of Fig. 1b). The state of each node is given by a three-dimensional vector 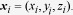 Details on the models can be found in the supplementary material. All nonzero weights 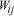 in the network on the top left are set to one and we choose the global coupling 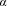 such that the nodes synchronize, that is, for any nodes 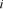 and 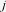 the difference of states 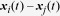 vanishes for 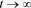. This synchronous dynamics can be seen in Fig. 1 for times 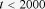. At time 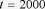 we add the new link 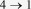 with a weight of 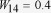, which leads to the strongly connected network on the top right. As can be seen for times 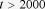 this destabilizes the synchronous state.

**Figure 3:**
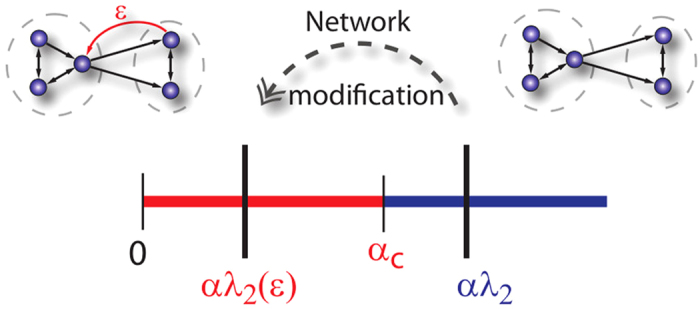
Change of the spectral gap. In a schematic representation we illustrate the motion of the spectral gap 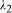 under structural modifications in case 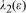 is real. The network on the right has a spectral gap such that 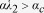. Adding a link as indicated decreases the gap to 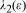, which violates the stability condition (3).

To understand this phenomenon, we analyze the stability of the synchronization subspace 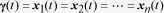, with 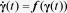. The variational equation of eq. (1) along 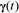 can be decomposed into 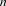 blocks of the form


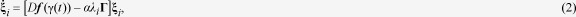


where 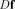 denotes the Jacobian, 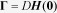. The 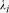 are the eigenvalues of the *Laplacian matrix* 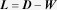 where 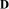 is the diagonal matrix with the row sums of 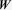 on its diagonal^16^,^17^. We assume that the spectrum of 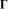 is real, which is the case for many applications such as electrical synapses and resistive coupling in electrical circuits. Now, the 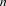 decoupled equations of the variational equation given by eq. (2) are 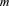-dimensional equations which only differ by the Laplacian eigenvalues 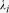. We consider the case where 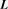 has a simple zero eigenvalue^5^. In this case, the eigenvalues of 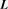 have nonnegative real parts, so we can order them increasingly according to their real parts 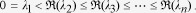 The eigenvalues with non-zero real part correspond to dynamics transverse to the synchronization subspace. Therefore, if the corresponding equations (2) have stable trivial solutions, synchronization in eq. (1) is stable. For a large class of coupling functions and local dynamics^16^, the stability condition for synchronization is given by


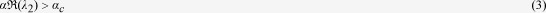


where 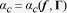. More involved stability conditions can be tackled, but the analysis becomes more technical without providing new insight into the phenomenon. Condition (3) shows that the *spectral gap* 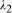 plays a central role for synchronization properties of the network. Structural changes which decrease the real part of 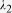 can destabilize the synchronous state, see Fig. 3.

Let us consider the generic case where the eigenvalue 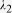 is simple. Using perturbation analysis^21^, we obtain the direction of growth of 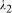 as a function of the structural modifications in the network. This analysis leads to a characterization of links which are capable of destabilizing the network. The spectral gap 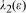 of a perturbed Laplacian 
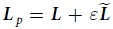
 is given by 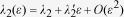 with


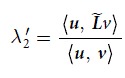


where 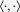 is the Euclidean inner product, 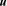 a left and 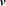 a right eigenvector of the unperturbed Laplacian 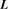 corresponding to the spectral gap 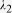, and we choose the vectors such that 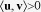.

If the network is undirected then 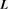 is symmetric and the left and right eigenvectors are dual. As 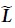 is positive semi-definite we obtain 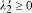. So increasing weights as well as adding links do not decrease the spectral gap in undirected networks^22^. This is an essential difference to *directed* networks.

Let us now consider a directed network composed of two strongly connected components as in the example in Fig. 1. Our approach is general and this choice is for the sake of simplicity. In this situation, the Laplacian can be represented in block form


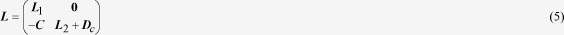


where 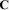 represents the cutset pointing from the strong component 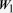 to 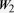, 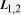 are the respective Laplacians and 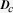 is again a diagonal matrix with the row sums of 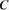 on its diagonal. As a consequence of the block structure, eigenvalues of 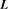 are either eigenvalues of 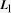 or eigenvalues of 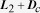. Suppose the eigenvalue with the smallest nonzero real part is located in the second component 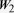 (this encloses the example from Fig. 1). Using a Perron-Frobenius argument, we can show that the eigenvalue 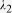 is real, see supplementary material. So, the eigenvectors 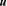 and 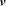 are real and by eq. (4) the motion of 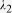 is along the real axis.

To determine the effects of the network changes on the spectral gap we investigate the left and right eigenvectors 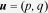 and 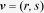. This decomposition corresponds to the triangular form of the Laplacian. Using the block structure, we can show that 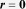 and that 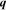 and 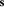 are left and right eigenvectors of 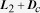. Furthermore, again by a Perron-Frobenius argument we can show that both 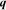 and 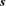 are positive. By our assumptions, 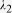 is not an eigenvalue of 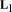. Therefore, we can solve the eigenvector equation for 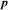 in terms of 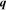 to obtain 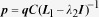, where 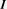 is the identity matrix.

Now we introduce a link in the opposite direction of the cutset, so the Laplacian writes as


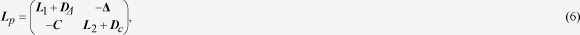


where 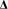 is the matrix describing the new link, and 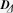 is the associated diagonal matrix, see supplementary material. This yields


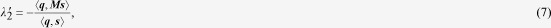


where


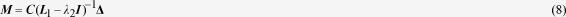


takes into account the structural changes 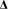. Now, such a modification will weaken the stability or even lead to instabilities if 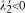. Determining the modifications that yield a decrease of the spectral gap is an involved problem, and we shall tackle it elsewhere. Here, we will focus on the example of Fig. 1, as it contains all central concepts without technical intricacies.

In the example from Fig. 1, 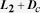 is symmetric and we have 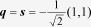. Moreover,


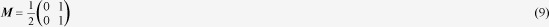


and 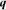 and 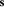 are eigenvectors of 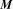 with eigenvalue 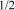. Because 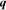 is a common eigenvector of both 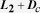 and 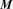 corresponding to a positive eigenvalue we obtain a decrease of the spectral gap with a rate 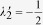. This is a main mechanism that generates instabilities: *the eigenvectors of* 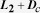 *lie in the space spanned by the eigenvectors of* 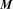 *with positive eigenvalues.*

Formally, we can obtain all the structural changes capable for destabilization as a function of the eigenvectors 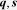 of 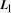 and 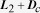. In contrast to undirected networks where there is a well developed theory relating eigenvectors to the underlying graph structure^23^, for directed graphs the theory is underdeveloped. Therefore, further analytical insights remain a challenge. From a computational point of view though, we can solve this problem for any given network.

For modifications 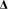 in the direction of the cutset the situation is clear, as eq. (4) reduces to


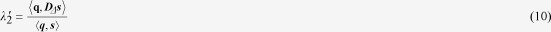


Because all the involved quantities are positive, the spectral gap does not decrease when reinforcing the cutset: it represents a *stabilising* class of links. By increasing strengths in the cutset we are guaranteed to enhance synchronization.

As we mentioned earlier, our findings are not restricted to the case of strong components connected by cutsets but can also be observed in strongly connected networks themselves as shown in Fig. 2. Here, all the links have weight one initially. When we increase the weight on link 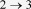 the spectral gap decreases and the synchronous state will become less stable. On the other hand, increasing weights on the links 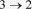, 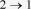 and 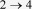 respectively increases the spectral gap. Furthermore, introducing the new links 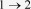 and 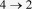 with a weak coupling strength also increases the spectral gap. As the network is strongly connected, the observed effects cannot be explained by simple master-slave behaviour. See supplementary material for simulations and more details on this network.

## Discussion

Our results revealed that directed and undirected networks behave essentially distinct under structural changes. Namely, assuming the stability condition eq. (3), synchronization loss caused by structural improvements is a property inherent to directed networks exclusively. If 
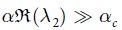
, the network modification may not destroy synchronization. However, it worsens the quality in the sense that the transient towards synchronization becomes larger. The network improvement can also affect the basin of attraction of the synchronization state^16^,^24^.

An important problem is to characterize the links capable of suppressing synchronization *a priori* in terms of their topological role in the network. This problem remains undisclosed. We provided a partial answer for a class of stabilizing links: the cutsets. However, our approach allows one to study such links with a low computational cost in terms of eq. (7).

Recently, interconnected networks have attracted much attention^25^,^26^, as they can exhibit catastrophic cascades of failures when connections are undirected. Our results suggest that interconnected networks in which interconnections are represented by (directed) cutsets behave qualitatively different. Furthermore, our results shed light on how to plan and design network modifications without destroying the network performance, as discussed for power-grids in^1^,^27^.

The catastrophic effects of structural improvements on the network function have a long history in game theory. In the realm of games such effects are known as Braess’s paradox^28^. In games, the effect occurs because the players take rational decisions to optimize their strategies. In the case of complex networks of dynamical systems considered here, the effect is dynamical and is a consequence of the motion of eigenvalues of the network Laplacian.

## Additional Information

**How to cite this article**: Pade, J. P. and Pereira, T. Improving Network Structure can lead to Functional Failures. *Sci. Rep.* **5**, 9968; doi: 10.1038/srep09968 (2015).
